# Comparison of data-merging methods with SVM attribute selection and classification in breast cancer gene expression

**DOI:** 10.1186/1471-2105-13-S7-S9

**Published:** 2012-05-08

**Authors:** Vitoantonio Bevilacqua, Paolo Pannarale, Mirko Abbrescia, Claudia Cava, Angelo Paradiso, Stefania Tommasi

**Affiliations:** 1 Department of Electrical and Electronics, Polytechnic of Bari, Via E. Orabona, 4, 70125 Bari, Italy; 2 Istituto Oncologico "Giovanni Paolo II", I.R.C.C.S Ospedale Oncologico di Bari, Viale Orazio Flacco 65, 70124 Bari, Italy

## Abstract

**Background:**

DNA microarray data are used to identify genes which could be considered prognostic markers. However, due to the limited sample size of each study, the signatures are unstable in terms of the composing genes and may be limited in terms of performances. It is therefore of great interest to integrate different studies, thus increasing sample size.

**Results:**

In the past, several studies explored the issue of microarray data merging, but the arrival of new techniques and a focus on SVM based classification needed further investigation. We used distant metastasis prediction based on SVM attribute selection and classification to three breast cancer data sets.

**Conclusions:**

The results showed that breast cancer classification does not benefit from data merging, confirming the results found by other studies with different techniques.

## Background

DNA microarray technology and expression profiles are the most suitable tools to investigate gene activity with respect to the progress of disease. Furthermore, they are useful for molecular classification of tumor types [1], for revealing complexity in the intrinsic cancer subtypes and for developing oncogenic pathway signatures as a guide to targeted therapies [2]. In particular, breast cancer has been extensively studied for gene expression in order to individualize a signature useful for molecular classification [3] and for prognostic purposes [4,5].

However, the sample size of each study is usually too small with respect to the number of the genes in analysis to allow an accurate statistical evaluation. Therefore, some authors used to analyze different data coming from different experiments with the goal of increasing sample size and thus increasing the power of the study.

This could be done in two ways: by meta-analysis [6,7], which is the statistical analysis of a large collection of results from individual studies for the purpose of combining their findings to reach a common result, or by data merging, which is analyzing all the raw data coming from different studies with similar biological questions together [8,9].

Typically, the first transformation applied to expression data, referred to as normalization and summarization, removes non-biological variability between arrays [10] and extracts gene level expression from probe intensities, respectively.

However, the transformation procedure cannot reduce completely the systematic differences from different data sets. When combining data sets from different experiments, non-biological experimental variation or ''batch effects'' are carried over and therefore it is inappropriate to combine data sets without adjusting for batch effects [11,12].

The goal of this paper is to compare the performance of various data merging methods. Our strategy for biological comparison is to use microarray data with known phenotypes associated with specific gene sets (pathways).

In literature, several techniques have recently been proposed for adjusting data for batch effects [13,14]. Many of these methods can only be applied to two batches at a time. In previous studies, merging data sets were applied to develop a robust gene signature prognostic of survival outcome discretized into two [15] or more categorical values, or diagnostic of tumor subtypes, or predictive of treatment response [16]. A comparison between several techniques to merge different datasets such as ComBat, Ratio_G, SVA, DWD, PAMR, has been carried out.

We used three breast cancer microarray data sets from three different studies in which all the samples came from lymph-node-negative patients who had not received adjuvant systemic treatment. We performed three pre-processing methods: Robust Multi-Chip Average [17], frozen RMA [18] and Quantile Normalization with Model-Based Expression Index [19]; subsequently we applied two data merging approaches: ComBat [20] and z-scoring standardization procedure for each dataset. frozenRMA is a recent method that performs batch effect removal inherently at summarization time and which has not yet been compared to other methods in an independent study. ComBat has been up to now the best performing method [21], and z-score is one of the first methods used to merge different datasets. Chen and others [22] suggest that the data from two experiments could be integrated for prognosis analysis after data standardization. The methods were compared from a new perspective, i.e. in terms of SVM classification and feature selection performances.

For microarray data classification the methodologies involving feature selection and classification applied to SVM are proposed in a previous study [23].

Variation attributable to batch effects before and after batch adjustment were identified using principal variation component analysis (PVCA) [24]. The success of batch effect removal was also evaluated using qualitative visualization techniques such as score plot of PCA and hierarchical clustering dendrogram.

Subsequently, the three microarray datasets, processed in different ways, were examined for specific patterns of pathway deregulation with respect to clinical disease outcome. For this reason we used the most popular gene-set analysis method, Gene Set Enrichment Analysis (GSEA) [25].

## Results

### Data merging validation

We directly merged the three microarray data sets, using the 22283 probe sets on Affymetrix HG-U133A microarray, to form an integrated data set in seven ways: RMA, Quantile/MBEI, RMA - ComBat, Quantile/MBEI-ComBat, RMA - Z-Score and Quantile/MBEI - Z-Score, frozenRMA. The integrated data set consisted of 111 samples with distant metastases and 394 samples free of distant metastases, randomly divided into training and testing sets but respecting the proportions of the complete dataset. We evaluated the classification performance using precision and recall of class 1 because of its clinical significance. Table 1 shows the results of the classifier which demonstrated less accuracy of data merging methods with respect to classification.

**Table 1:** Classification performance

CB-RMA				ZS-RMA			
Validation		Recall	Precision	Validation		Recall	Precision

LDOCV	FK	0.568	0.263	LDOCV	FK	0.622	0.277
	GSE2990	0.5	0.286		GSE2990	0.536	0.3
	GSE11121	0.63	0.315		GSE11121	0.609	0.322
	Average	0.576	0.289		Average	0.596	0.301
LOOCV		0.631	0.370	LOOCV		0.512	0.317
Percentage fold		0.531	0.273	Percentage fold		0.563	0.228
Stratified perc. fold		0.677	0.198	Stratified perc. fold		0.613	0.25

**RMA**				**CB-MBEI**			

Validation		Recall	Precision	Validation		Recall	Precision

LDOCV	FK	0.622	0.304	LDOCV	FK	0.649	0.282
	GSE2990	0.607	0.283		GSE2990	0.571	0.314
	GSE11121	0.609	0.280		GSE11121	0.652	0.341
	Average	0.613	0.289		Average	0.631	0.313
LOOCV		0.613	0.289	LOOCV		0.550	0.289
Percentage fold		0.5	0.208	Percentage fold		0.625	0.282
Stratified perc. fold		0.645	0.257	Stratified perc. fold		0.692	0.237

**ZS-MBEI**				**MBEI**			

Validation		Recall	Precision	Validation		Recall	Precision

LDOCV	FK	0.73	0.297	LDOCV	FK	0.486	0.290
	GSE2990	0.571	0.333		GSE2990	0.643	0.290
	GSE11121	0.696	0.36		GSE11121	0.522	0.414
	Average	0.677	0.331		Average	0.539	0.339
LOOCV		0.550	0.279	LOOCV		0.550	0.299
Percentage fold		0.625	0.299	Percentage fold		0.625	0.282
Stratified perc. fold		0.692	0.225	Stratified perc. fold		0.808	0.28

**fRMA**							

Validation		Recall	Precision				

LDOCV	FK	0.649	0.279				
	GSE2990	0.464	0.283				
	GSE11121	0.5	0.319				
	Average	0.544	0.307				
LOOCV		0.512	0.308				
Percentage fold		0.588	0.23				
Stratified perc. fold		0.581	0.265				

### Data merging verification

Batch effects are present in microarray experiments when data are combined from different studies. To assess the quantity of batch effect and to compare the data merging methods Combat, ZScore and fRMA, we performed a novel hybrid approach known as principal variance component analysis.

Principal Variance Component Analysis (PVCA) integrates two methods: principal components analysis (PCA) finds low dimensional linear combinations of data with maximal variability and variance components analysis (VCA) attributes and partitions variability into known sources via a classical random effects model.

The first step is to obtain the covariance/correlation matrix of the microarray expression data matrix. Secondly, PCA is applied to the correlation matrix and once the number of principal components is determined, a variance component model is fitted separately to each of these principal components. The variation in each principal component is weighted by its eigenvalue from PCA, and the resulting value represents the overall variation explained by that component.

**Figure 1:**
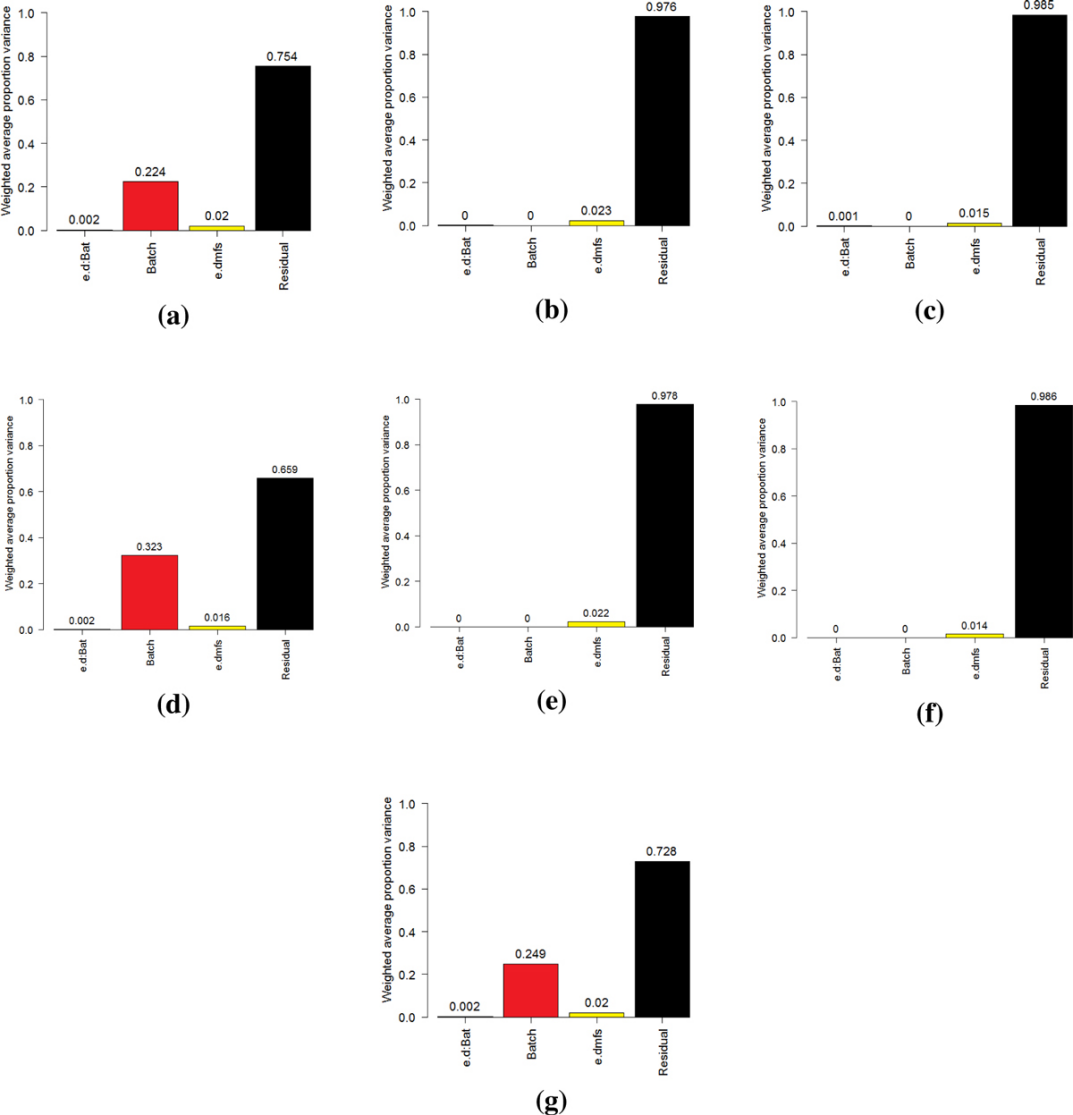
**PVCA results**. RMA data (a), RMA - ComBat data (b), RMA - Z-Score data (c), MBEI data (d), MBEI - ComBat data (e), MBEI - Z-Score data (f), fRMA data (g).

The PVCA revealed that batch effects explained 22.4% of the overall variation in the RMA data (Figure 1a) and 32,3% in the Quantile/MBEI data (Figure 1d).

After applying ComBat (Figure 1b,e) and Z-Score (Figure 1c,f) the variation was completely eliminated. The worst performance seemed to be that of fRMA (Figure 1g) which showed a 24,9% threshold of variation of the batch effects.

**Figure 2:**
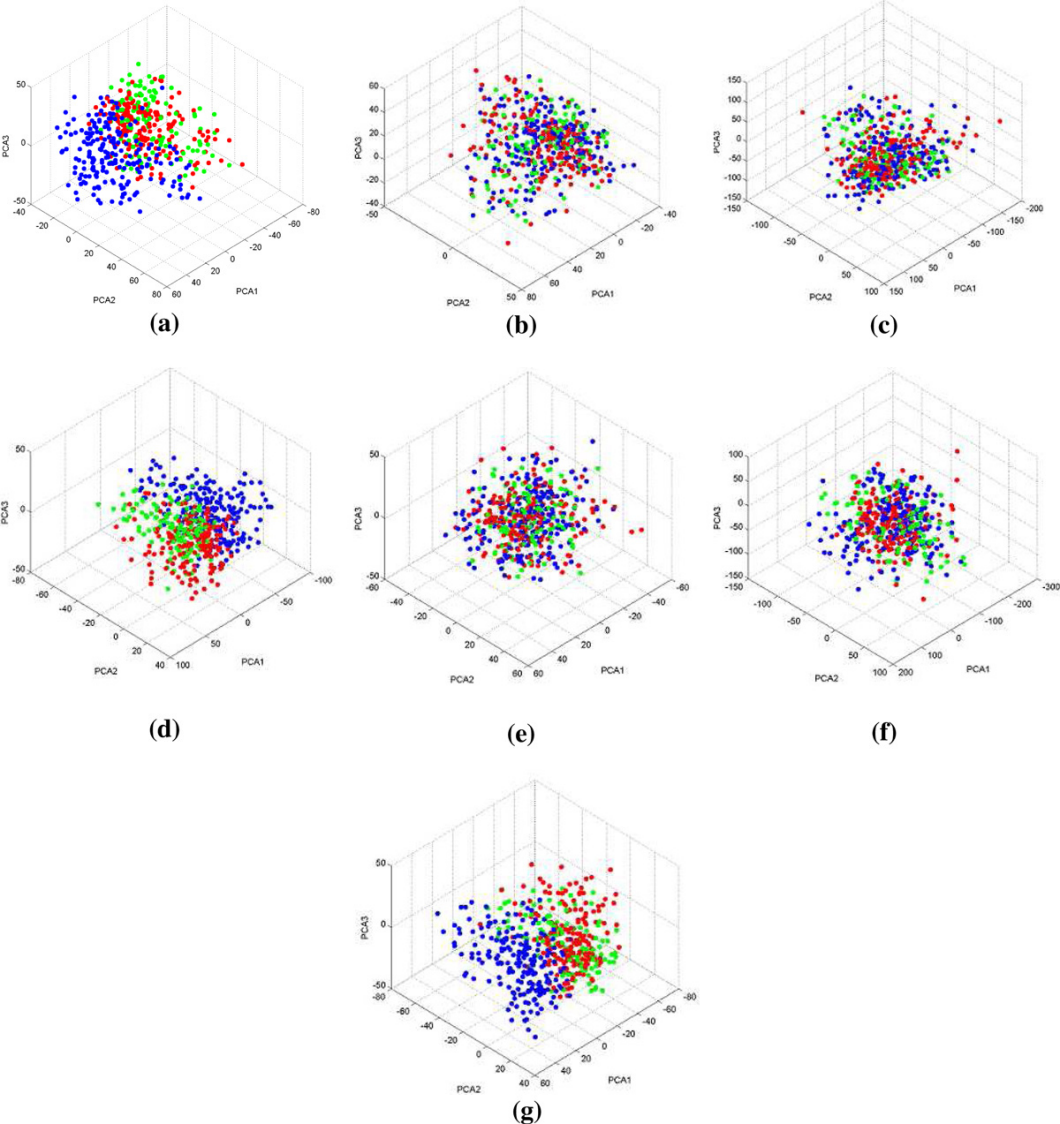
**PCA results**. RMA data (a), RMA - ComBat data (b), RMA - Z-Score data (c), MBEI data (d), MBEI - ComBat data (e), MBEI - Z-Score data (f), fRMA data (g); FK - NCC samples in red, GSE2990 samples in green, GSE11121 samples in blue.

To assess the removal of microarray bias effect across data sets, Principal Component Analysis (PCA) was applied to the data sets after the application of data merging methods. The aim was to reveal intermixing of samples from different datasets before and after adjustment. The results of these approaches (Figure 2) demonstrate that samples referring to the same dataset cannot be grouped after using merging methods. This trend was respected in the RMA - ComBat data (Figure 2b), RMA - Z-Score data (Figure 2c), MBEI - ComBat data (Figure 2e) and MBEI - Z-Score data (Figure 2f), as shown in the graphs of the first three principal components. Conversely it is possible in the RMA data (Figure 2a), MBEI data (Figure 2d) and fRMA data (Figure 2g).

Again, fRMA seemed to be unable to adjust the combination among the three datasets.

**Figure 3:**
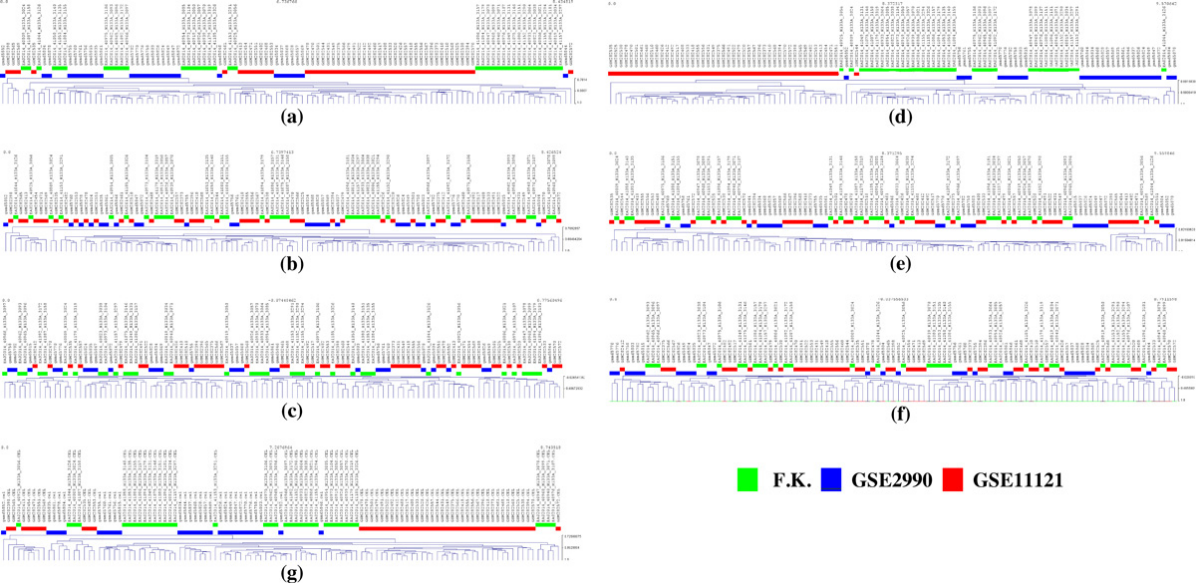
**Hierarchical clustering before and after batch adjustment**. Each color marks a sample as belonging to a different batch. RMA data (a), RMA - ComBat data (b), RMA - Z-Score data (c), MBEI data (d), MBEI - ComBat data (e), MBEI - Z-Score data (f), fRMA data (g) are shown.

The results of hierarchical clustering analyses of class 1 before and after batch adjustments are presented in Figure 3. The sample clustering showed a separation of the three groups of samples where adjustment for batch effects was not performed and the samples were only summarized by RMA (Figure 3a) and MBEI (Figure 3d). The distinct clustering of the tumor groups did not indicate a large difference in gene expression patterns but only a bias introduced by experimental conditions. We clearly identified a cluster of samples performed in the same batch without any clear biological interpretation.

After batch adjustment of the RMA dataset (Figure 3b-c) and the MBEI dataset (Figure 3e-f), the clusters were no longer confused with the batch effects. The EB adjustment (Figure 3b,e) has the advantage of being robust to outliers in small sample sizes, thus no notable difference was seen in our experimental set-up.

The GSE1121 samples clustered together more sharply than the GSE2990 and the Foekens samples which created overlapping clusters.

Normalized Mutual Information (NMI) [26] between the distribution of class labels and the distribution of cluster labels was used to assess the results of clustering after cutting the tree at a given threshold (0.5). A value of 0.69 was observed for the RMA dataset, which decreased to 0.23 and 0.24 after batch adjustment. The NMI score was higher for the MBEI dataset (0.82), again falling to 0.24 and 0.27 after batch adjustment. For fRMA we found a score of 0.73.

### Gene analysis

As previously described, two separated methods, SAM and RFE, were used to identify differentially expressed probes and to show the validation of RFE as feature selection in the training step of the classification.

SAM resulted in 204, 289, 166, 197, 257, 197 and 210 differentially expressed genes for the RMA, CB-RMA, ZS-RMA, MBEI, CB-MBEI, ZS-MBEI and fRMA respectively.

Table 2 summarizes the results of shared genes extracted by SAM and RFE - SVM.

**Table 2:** List of shared probes extracted by SAM and RFE-SVM

Probe set	RMA	MBEI	CB RMA	CB MBEI	ZS RMA	ZS MBEI	Probe set	RMA	MBEI	CB RMA	CB MBEI	ZS RMA	ZS MBEI
200944_s_at	-	-	-	-	x	-	210084_x_at	-	-	-	x	-	x
201236_s_at	-	-	x	-	-	-	210098_s_at	-	-	-	-	-	x
201340_s_at	x	-	x	-	x	-	211776_s_at	-	-	x	-	-	-
201602_s_at	-	-	x	-	-	-	211868_x_at	-	-	-	x	-	x
202072_at	-	-	-	-	x	-	212327_at	-	-	-	-	-	-
202464_s_at	x	x	x	x	-	x	212763_at	-	-	-	-	x	-
202496_at	-	-	-	-	x	-	212840_at	x	-	x	-	-	-
203413_at	-	-	x	-	-	-	213502_x_at	-	x	-	x	-	-
204444_at	-	-	-	x	-	-	214087_s_at	-	x	-	x	-	-
204540_at	x	-	x	-	-	-	214669_x_at	-	-	-	-	-	x
204932_at	-	-	-	x	-	-	214766_s_at	-	-	x	-	x	-
205242_at	-	x	-	x	-	-	215382_x_at	-	-	-	-	-	-
205898_at	x	-	-	-	-	-	217023_x_at	-	-	-	-	-	-
206143_at	x	-	-	-	-	-	217766_s_at	-	-	-	-	-	x
206710_s_at	-	-	x	-	-	-	218009_s_at	-	x	-	-	-	-
207118_s_at	-	-	-	-	x	-	218542_at	-	-	x	-	-	-
207134_x_at	-	-	-	-	-	-	219004_s_at	x	-	-	-	-	-
207741_x_at	-	-	-	x	-	x	219115_s_at	-	x	-	x	-	-
208080_at	-	-	-	-	x	-	219148_at	-	-	-	-	-	-
208577_at	-	-	-	-	x	-	219296_at	-	-	-	-	-	-
208696_at	-	-	-	x	-	-	219306_at	-	-	-	-	-	-
209172_s_at	-	-	-	x	-	-	219438_at	-	-	x	-	-	-
209380_s_at	x	-	x	-	x	-	219743_at	-	-	-	x	-	x
209448_at	-	-	x	-	x	-	219990_at	x	-	x	-	-	-
209869_at	-	x	-	x	-	x	220177_s_at	-	-	x	-	-	-

A total of 50 common probes were found from SAM and RFE comparison. Most probes were successfully identify by RMA-Combat. None was found by fRMA.

These results were analyzed by fisher's exact test to examine the significance of overlapping between the two gene selection algorithms. After correction for multiple testing by the Benjamini-Hochberg standard false discovery rate correction, we found that it could not be excluded that the overlappings were derived by chance for all but the fRMA dataset, where the test was positive for the "less" alternative. This result indicates that the frozen RMA normalization hindered RFE from finding biologically relevant genes, although SAM reported a number of genes comparable to the other normalization methods. With the "greater" alternative, every merging technique showed a pvalue >> 0.05, showing that the number of overlapping genes between FRE and SAM is never greater than expected by chance.

### Enrichment analysis

We explored the performance of various pre-processing and data merging techniques by using biological pathway-based analysis and determining whether a group of differentially expressed genes is enriched for a particular set.

Here, we utilized a powerful analytical method called Gene Set Enrichment Analysis (GSEA) focusing on several biological pathways: EGF, Stathmin, HER2, BRCA1,Homologous Recombination, which are associated with breast cancer progression.

The use of different methods of data merging and preprocessing can lead to a problem of poor congruency among datasets. To compare the ability of the different methods we performed GSEA algorithm showing the heat map, enrichment scores and corresponding p-values [27].

All probe sets were pre-ranked using SNR (signal to noise ratio) with respect to their correlation with distant metastasis-free survival.

The signal to noise ratio (SNR) detects the expression patterns with a maximal difference in mean expression between two groups and minimal variation of expression within each group.

The order probe set list was used as the GSEA input for pathway analysis.

**Figure 4:**
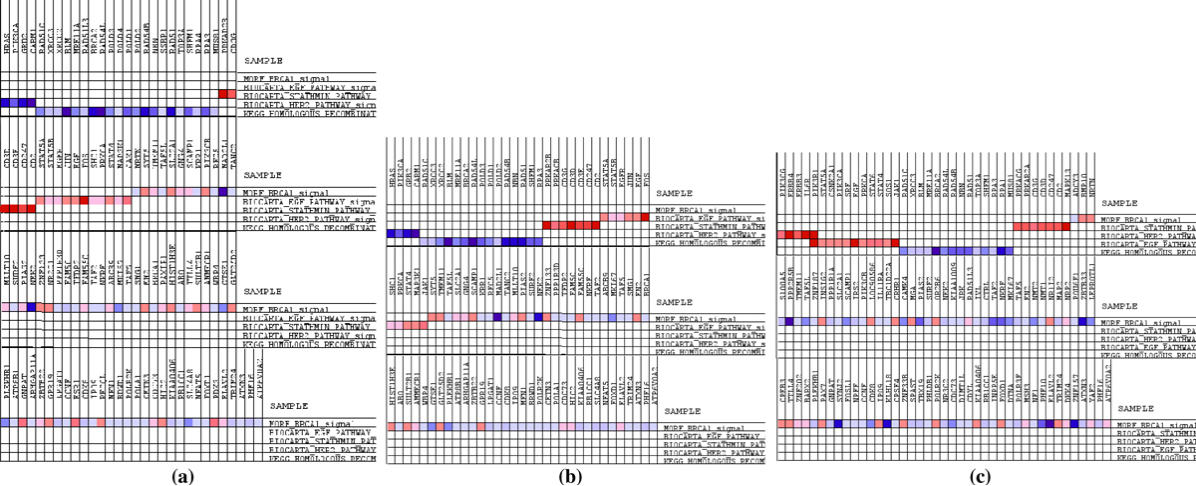
**Heat map of RMA pre-processed datasets**. RMA data (a), RMA - ComBat data (b), RMA - Z-Score data (c).

**Figure 5:**
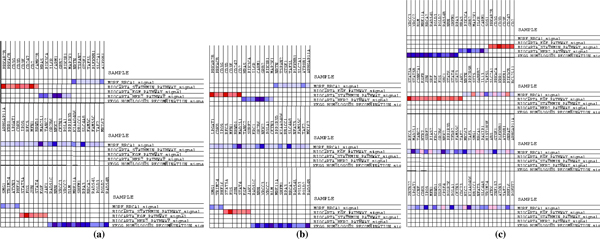
**Heat map of MBEI pre-processed datasets**. MBEI data (a), MBEI - ComBat data (b), MBEI - Z-Score data (c).

**Figure 6:**
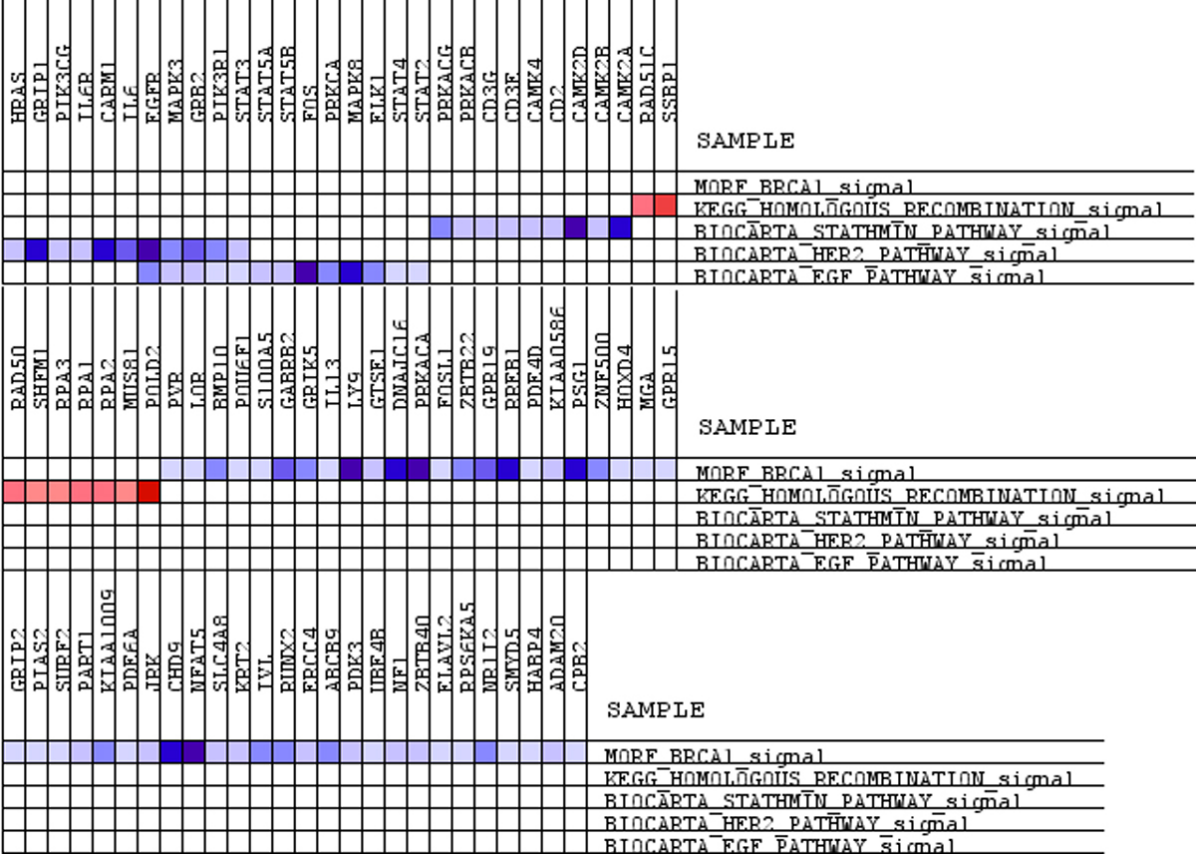
**Heat map of fRMA merged datasets**.

In a heat map, expression values are represented as colors, where the range of colors (red, pink, light blue, dark blue) shows the range of expression values (high, moderate, low, lowest). The light blue and dark blue bars reflect genes that are positively associated with DMFS (Disease Metastasis Free Survival), indicating a higher expression in tumors without metastatic capability. The red bars reflect genes that are negatively associated with DMFS, indicative of higher expression in tumors with metastatic capability. The heat map of data sets that are pre-processed by robust multi-array average (RMA) and MBEI and merged by fRMA are presented in Figures 4, 5, 6.

Considering the expression level of the genes reported in each considered pathway, it can be observed that applying ComBat after RMA (Figure 4b) or MBEI (Figure 5b) leads to similar results to RMA (Figure 4a) and MBEI (Figure 5a) alone, respectively. However, the Z-Score method reported activation of different genes in each pathway after both methods. (Figure 4c; 5c).

The heat map of RMA and MBEI data identified two over-regulated gene sets, EGF and Stathmin Pathway, indicative of higher expression in tumors with metastatic capability and three up-regulated gene sets, Homologous Recombination, BRCA1 and HER2 pathway, indicating a higher expression in tumors without metastasis.

Gene Set Enrichment Analysis computes an enrichment score (ES) for a given gene set which reflects the degree to which a gene set is over-represented at the extremes of the entire ranked list of genes.

A positive ES suggests gene set enrichment at the top of ranked list; a negative ES suggests gene set enrichment at the bottom of the ranked list.

The nominal p-value estimates the statistical significance of the enrichment score for a single gene set.

We found (Tables 3, 4) that both RMA and MBEI detected Homologous Recombination as the most significant gene set, however, the statistical significance of RMA of this gene set was much greater, p= 0.0082 (RMA) p = 0.0073 (CBRMA) p = 0.0019(ZSCORE) versus p = 0.0057 (MBEI) p = 0.0020 (CBMBEI) p = 0.3836 (ZSMBEI).

**Table 3:** Application of GSEA to the different RMA and fRMA pre-processing and data merging methods

Gene set	RMA		CB RMA		ZS RMA		fRMA	
	**ES**	**p-value**	**ES**	**p-value**	**ES**	**p-value**	**ES**	**p-value**

EGF pathway	-0.4229	0.1285	-0.4542	0.0923	-0.4127	-0.3109	-0.3731	0.3145
Stathmin pathway	-0.4307	0.4712	-0.4281	0.5079	-0.2711	-0.6063	-0.2844	0.9051
Homologous recombination	0.6766	**0.0082**	0.6956	**0.0073**	0.6962	**0.0019**	0.3378	0.5750
BRCA1 pathway	0.2974	**0.0278**	0.3017	**0.0403**	0.2982	0.1353	-0.1768	0.8875
HER2 pathway	0.3388	0.5658	0.3475	0.5507	0.4504	0.0730	-0.3795	0.4612

**Table 4:** Application of GSEA to the MBEI pre-processed datasets

Gene set	MBEI		CB MBEI		ZS MBEI	
	**ES**	**p-value**	**ES**	**p-value**	**ES**	**p-value**

**EGF pathway**	-0.4793	**0.0451**	-0.4387	0.0647	-0.2616	0.6179
**Stathmin pathway**	-0.3323	0.5778	-0.3492	0.5848	-0.3551	0.5186
**Homologous recombination**	0.6516	**0.0057**	0.6533	**0.0020**	-0.3582	0.3836
**BRCA1 pathway**	0.3067	0.0746	0.3032	0.0763	0.3112	0.1509
**HER2 pathway**	0.3434	0.5098	0.3747	0.3727	0.38071	0.2003

Overall, GSEA with RMA normalization identified two gene sets, Homologous Recombination and BRCA1 pathway, as statistically significant at p<0.05 whereas GSEA with MBEI normalization detected Homologous Recombination and EGF pathway with the same p-value.

For all gene sets, p-values obtained by RMA normalization were generally smaller than the p-values of corresponding gene sets obtained by MBEI normalization.

With Combat data, GSEA identified, although with higher significance, the same gene sets derived from RMA or MBEI data alone.

Comparison of different data merging techniques confirmed that ZScore shows more variation than MBEI or RMA data alone.

**Figure 7:**
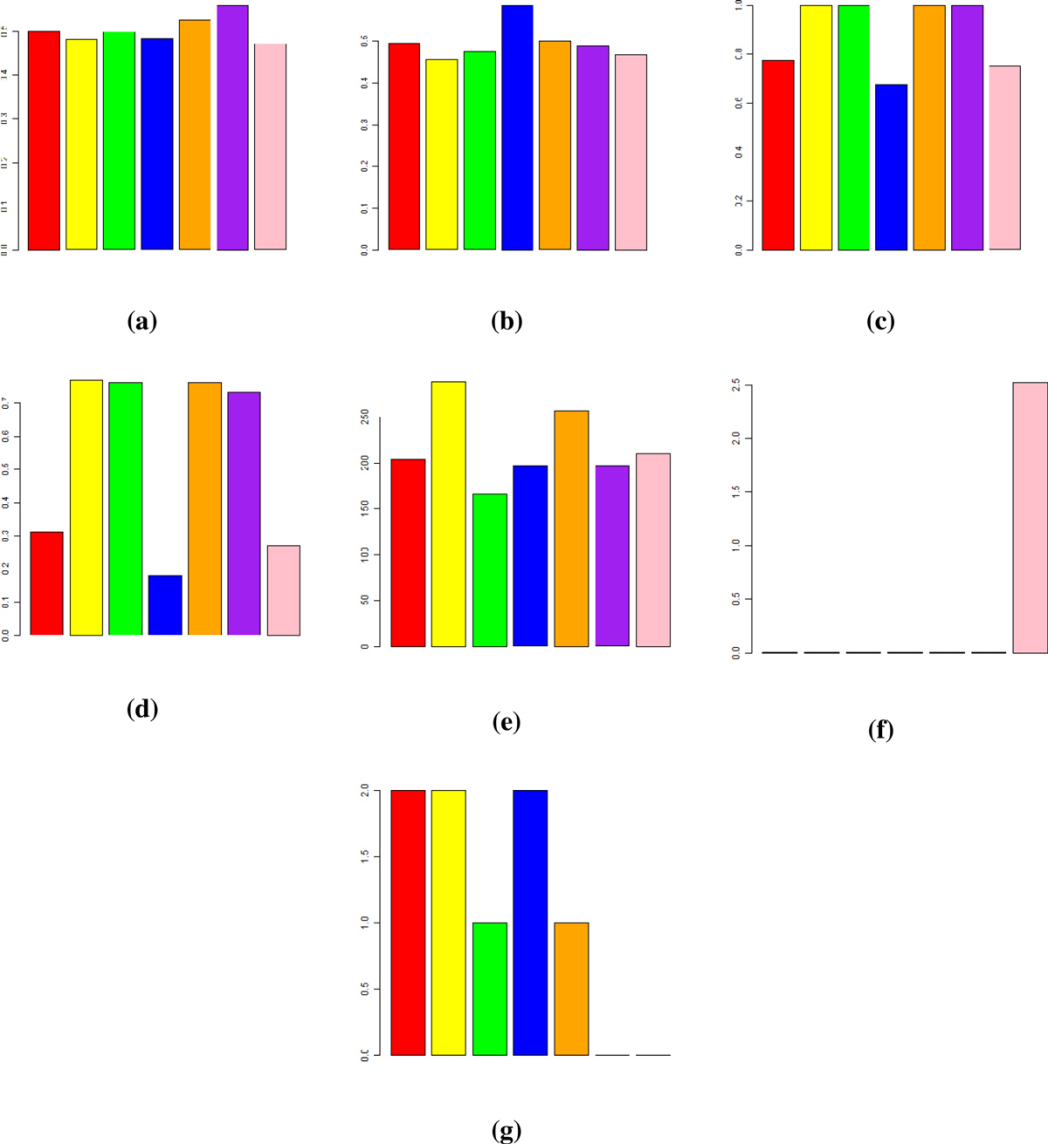
**Overview of results**. An overview of the results of the data merging evaluation methods used in this study for SVM classification with leave-dataset-out (a) and stratified percentage folding (b), PVCA analysis (c), clustering (d), SAM (e), RFE-SAM overlapping (f) and enrichment analysis (g). The bars in each plot refers to RMA (red), CB-RMA (yellow), ZS-RMA (green), MBEI (blue), CB-MBEI (orange), ZS-MBEI (purple) and fRMA (pink) respectively.

fRMA revealed different gene sets regulated with a higher p-value than ZScore and Combat. A comparative representation of the different evaluation and verification methods is presented in Figure 7. For the classification analysis an F-measure with beta equal to 2 is reported. This measure gives a recall higher than precision and is more suitable for a summarization of the classifier performances in the clinical context. For the PVCA analysis the weight of the batch effect component is reported. Figure 7 also presents a complement to 1 of the NMI score for the clustering analysis, the number of relevant genes found by SAM and the -log10(pvalue) for the negative effect of the merging algorithm on the ability of RFE to select biologically relevant genes. The number of enriched pathways found by GSEA is shown at the end.

## Discussion

In this study we have presented the results of the classification of breast cancer microarray data, with respect to the event of distant metastasis free survival in terms of recall and precision. The results show very low classification performances. This is explained in part by heterogeneity of the data in terms of tumor grade, tumor size, histopathological tumor type and progesterone and estrogen receptor that might negatively influence the prediction. In the past, different classifiers were built for patients showing different values for these features with successful results [28].

The F-2 scores in Figure 7a show that for both z-score and Combat merging procedures the results of classification with LDO validation are enhanced only when applied to the MBEI summarization.

Z-score performed better than Combat with both summarization techniques and the worst results were achieved by fRMA.

The LDO validation is representative of a clinical application where batches of patients are analyzed after the classifier has been built on previous batches. In this context of practical interest the merged datasets show some advantages compared to non-merged datasets.

Figure 7b shows however that these results are comparable to those of non- merged MBEI datasets with stratified percentage folding, where obviously the classifier cannot benefit from the higher number of samples coming from different batches and the results could have been even better if the classifier had been built on a single batch. In this case the classifier probably separated the different batches in different hyperplanes. Thus we can conclude that the SVM classifier could not benefit from the merging of different batches and the increasing number of samples.

The difference between Stratified Percentage Fold and weighted LDOCV average is very sharp in the MBEI pre-processed datasets showing that this method gets greater benefits from the merging algorithms. The comparison within the RMA processed datasets shows that the non-merged dataset has even higher performances than the ComBat and Z-score merged ones in stratified percentage folding.

However, the merging procedure achieved some results, as shown by the PVCA and Clustering analysis, where both z-score and Combat datasets behaved better than non- merged ones. Combat and z-score did not show sharp differences with these techniques. When comparing the differences in the SVM versus PVCA/Clustering results, we can conclude that the merging algorithms are an effective tool, but should be handled with care when it comes to the next algorithm in the workflow pipeline.

fRMA showed performances comparable to non-merged datasets also in the PVCA/Clustering case and our findings exclude it from being a good data merging algorithm even when compared with the simplest strategy of z-scoring.

A comparative study of performances of Recursive Feature Elimination (RFE) with SAM has been given as an additional evaluation tool for the validation of RFE as feature selection.

Also in this case we found an exception: SAM did not suffer from the fRMA normalization and reported a number of differentially expressed genes compared to the other techniques. Again in this case Combat outperformed z-score with a number of genes almost double.

In the same step of gene selection, RFE showed results markedly different from SAM, and the number of common genes, equal to zero, is not derived from chance: fRMA hindered RFE from finding biologically relevant genes.

The biological meanings of gene expression have been also analyzed in terms of enrichment analysis.

Our objectives tested predefined gene sets for their association with breast cancer progression, assuming that gene expression changes can be identified at the level of co regulated gene sets rather than individual genes.

The application of gene set analysis by GSEA was also useful in finding common biological pathway changes and in comparing different preprocessing and data merging methods.

GSEA detected three gene sets, Homologous Recombination, BRCA1 and HER2 pathway, as significantly up-regulated versus two gene sets, EGF and Stathmin Pathway, which were over-regulated; although in many cases the same gene sets were identified as significant by various programs, in a direct comparison across MBEI and RMA normalization using breast cancer datasets, the p-values of RMA were lower than the respective p values obtained by MBEI (Table 4), indicating that RMA is more statistically sensitive than MBEI for these gene sets.

Again, fRMA found gene sets with lower significance than the others and Combat outperformed z-score, although the latter showed better results in terms of classification performances. In terms of pathway enrichment, the results of Combat merging are comparable to those of non-merged datasets.

Overall, this study confirms the previous studies that did not show benefits of survival prediction with merged datasets as compared to individual data sets using linear methods [29]. It also confirms that Combat outperformed the other programs and the different pre-processing methods (MBEI and RMA) did not show significant variation giving rise to improvement of prediction. [21].

Furthermore, the analysis of gene expression level in specific pathways confirmed the better performance of ComBat with respect to Z-Score, and focusing on PVCA, fRMA was inaccurate in removing the batch effect from the data. This is evident when observing the graphical representation of data after PCAnalysis, hierarchical clustering and PVCA.

## Conclusions

The present study showed the difficulty of merging data from different datasets, even if they come from the same type of chip, due to the low accuracy even when using different approaches. This suggests that because of the low recall and precision of all methods, data merging does not seem to be the elective approach to combine samples in array analysis. Thus a better way to improve accurate signature from microarray datasets is to apply a meta-analysis rather than merging all raw data or developing algorithms that can leverage the effect of data merging, since we have demonstrated that it is algorithm-dependent.

## Methods

### Dataset and preprocessing methods

**Figure 8:**
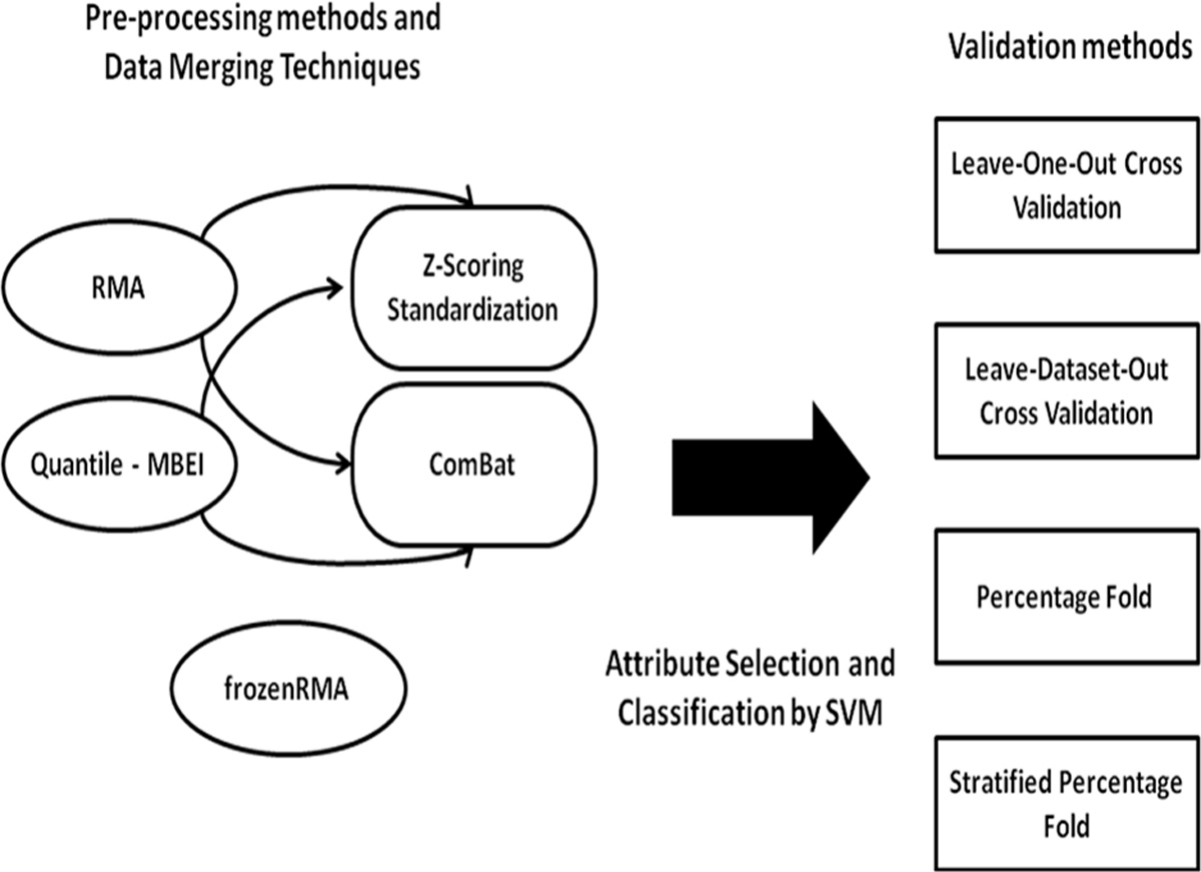
**Study workflow**.

We applied pre-processing and data merging techniques to three breast cancer data sets: GSE11121, GSE2990 (Gene Expression Omnibus) and a dataset used by Foekens et al. in [30], containing respectively 200, 125 and 180 samples from the same Affymetrix GeneChip Human Genome U133A platform. All of the patients in the data sets had lymphonode-negative tumors and did not receive adjuvant systemic treatment. The design of the study is reported in Figure 8.

Three pre-processing methods were applied to microarray data to compute expression values from input CEL files: Robust Multi-array Average (RMA), Quantile normalization - Model-Based Expression Index (MBEI) and frozenRMA. This last method estimates batch effect directly from probe intensities. The other methods were integrated in an independent manner with two data merging techniques: 1) ComBat 2) Z-score. All computations were done using the free statistical software package R and DNA-Chip Analyzer (dChip).

Processing Affymetrix expression arrays usually consists of three steps: background adjustment, normalization and summarization and, in this study, the data sets were processed in three ways (Table 5).

**Table 5:** Pre-processing methods

Preprocessing			
**Method**	**Background correction**	**Normalization**	**Summarization**

RMA	Global correction from posterior mean given the observed PM	Quantile: intensities quantiles Average	Linear model including array and probe effects fitting by median polish
Quantile -MBEI	-	Quantile: baseline intensities	Model assuming multiplicative probe effect and additive error
FrozenRMA	RMA-like	Quantile: reference distribution intensity	RMA-like including batch effect in the model

Before proceeding to the next analysis, it is necessary to detect and remove the batch effects. Here, the term "batch effect" refers to experimental variations of datasets generated by different labs.

An Empirical Bayes method, called Combating Batch Effects when Combining Batches of Gene Expression Microarray Data (ComBat) and Z-Score were used to adjust the systematic difference of differently normalized data generated by the three different labs. The algorithm of Johnson et al. was implemented into the software package ComBat, which includes two empirical Bayes frameworks, one with parametric and one with non-parametric approach. With this algorithm, location and scale model parameters are specifically estimated by pooling information across genes in each batch to adjust for the batch effect parameter estimates toward the overall mean of the batch estimates.

Each data set was alternatively standardized using a simple z-score transformation method and combined for analysis. In contrast to classic pre-processing and data merging techniques, we applied another pre-processing algorithm, Frozen Robust Multiarray Analysis (fRMA) which allowed us to analyze microarray in batches and then combine the data for analysis. fRMA without further adjustment for batch effects is similar to RMA when the data are analyzed as a single batch.

This procedure led to seven differently-merged datasets: RMA without merging (RMA), RMA data merged by Combat (CB-RMA) and by z-score (ZS-RMA), MBEI without merging (MBEI), MBEI data merged by Combat (CB-MBEI) and by z-score (ZS-MBEI) and fRMA data (fRMA).

### Classification

After these steps, the above procedures were analyzed to extract a gene signature, in order to associate it with distant-metastasis-free survival (dmfs) discretized into two values (0,1). The datasets were unbalanced towards class 0, but from a clinical stand point, the cost of false negatives is higher than false positives: patients that should receive treatment may be neglected and not receive treatment. For this reason, the class 0 set were down-sampled until half spread.

The training step applies a SVM based Recursive Feature Elimination (RFE) method [31,32] for the selection of genes using an iterative procedure which ranks features and discards the worst one, after sub sampling of the dataset to make the distribution size of the two classes equal. This model, composed of 400 gene expression levels, was then used for the training of a linear support vector machine, a classifier widely used in bioinformatics for its features [33]. The number of genes was set to 400 after a comparison of results for each dataset. The classifier performances were optimal around this value and relatively insensitive to small variations in the number of features. The next step consisted of testing the dataset that had not been used on the samples to train the classifier. In this study, we used two performance measures of the classifier: recall (sensitivity) and precision.

### Validation methods

The models were validated using Leave-One-Out Cross Validation (LOOCV), Leave-Dataset-Out Cross Validation (LDOCV), Percentage Fold and Stratified Percentage Fold. The Leave-One-Out (or LOO) is a simple cross-validation. Each learning set is created by using all the samples except one, the test set being the sample left out.

Leave-one-out cross validation is used in the field of machine learning to determine how accurately a learning algorithm will be able to predict data that it was not trained on. It creates all the possible training/test sets by using a single observation from the original sample as the testing data, and the remaining observations as the training data. LDOCV is very similar to LOOCV but it considers the single dataset rather than samples. Percentage Fold randomly divides the merged data set into training and testing sets, 66% and 34% respectively; the Stratified Percentage Fold splits each dataset by preserving the same percentage for training and testing sets.

### Principal variation component analysis (PVCA) and hierarchical clustering

In this study we utilized PVCA to compute non biological experimental variation or "batch effects" carried over when we combined the three data sets from different experiments. The approach utilizes two data analysis methods: first, principal component analysis (PCA) is used to efficiently reduce data dimension while maintaining the majority of the variability in the data, and variance component analysis (VCA) fits a mixed linear model using factors of interest as random (or batch) effects and other variables (or covariates) to estimate and partition the total variability.

Average linkage hierarchical cluster analysis was carried out using Mev software with a modified Pearson correlation as a similarity metric. The clustering on each dataset was performed using all the genes in the platform.

### Gene and enrichment analysis

The genes that exhibited little variation in their profile and which were not of interest were removed from data for the subsequent pathway analysis. Filtered datasets were also analyzed for statistically significant genes using the Significance Analysis of Microarray (SAM) algorithm [34]. SAM is a statistical technique for determining whether changes in gene expression are statistically significant. The analysis was performed using MeV (MultiExperimentViewer) [35] to search for genes that correlated with distant metastasis.

A comparative study of performances of Recursive Feature Elimination (RFE) with SAM was carried out as an additional evaluation tool.

For further validation of different data merging and processing technique comparison, we analyzed specific pathways associated with breast cancer progression: EGF, Stathmin, HER2, BRCA1 and Homologous Recombination. The pathway database was compiled from the Kyoto Encyclopedia of Genes and Genomes (KEGG) and Biocarta. The GSEA algorithm, an established method in pathway enrichment analysis, was used. Enrichment analysis is an automated statistical technique to analyze and interpret large gene lists using a priori knowledge. The strength of gene regulation information is an important component of cancer classifiers, which have been proven to be able to leverage such information [36]. A data merging algorithm that maximizes the regulation information contained in the dataset is also suitable for providing an optimal substrate for classification.

The genes can be ordered in a rank list, according to their differential expression between the classes, and the goal of GSEA algorithm is to determine whether the members of specific pathways are randomly distributed throughout the ranked list or primarily found at the top or bottom. If a statistically significant number of genes from a specific pathway is present in the gene list, it may indicate that the biological pathway plays a role in the biological condition under study.

## Competing interests

The authors declare that they have no competing interests.

## Authors' contributions

VB, PP, MA and CC carried out the data merging and classification, clustering and SAM. VB, MA and CC carried out the PVCA, PCA and enrichment analysis. VB, ST, AP and PP conceived of the study and participated in its design and coordination. VB, PP, MA, CC and ST drafted the manuscript and then all the authors read and approved the final manuscript.

## References

[B1] Gatza ML Lucas JE Barry WT Kim JW Wang Q Crawford DM Datto BM Kelley M Mathey-Prevot B Potti A Nevins JR A pathway-based classification of human breast cancer Proc Natl Acad Sci USA 2010 107 6994 6999 10.1073/pnas.0912708107 20335537 PMC2872436

[B2] Bild AH Yao G Chang JT Wang Q Potti A Chasse D Joshi M-B Harpole D Lancaster JM Berchuck A Olson JA Marks JR Dressman HK West M Nevins JR Oncogenic pathway signatures in human cancers as a guide to targeted therapies Nature 2006 439 353 357 10.1038/nature04296 16273092

[B3] Sørlie T Perou CM Tibshirani R Aas T Geisler S Johnsen H Hastie T Eisen MB van de Rijn M Jeffrey SS Thorsen T Quist H Matese JC Brown PO Botstein D Lønning PE Børresen-Dale A-L Gene expression patterns of breast carcinomas distinguish tumor subclasses with clinical implications Proc Natl Acad Sci USA 2001 98 10869 10874 10.1073/pnas.191367098 11553815 PMC58566

[B4] van de Vijver MJ He YD van't Veer LJ Dai H Hart AAM Voskuil DW Schreiber GJ Peterse JL Roberts C Marton MJ Parrish M Atsma D Witteveen A Glas A Delahaye L van der Velde T Bartelink H Rodenhuis S Rutgers ET Friend SH Bernards R A gene-expression signature as a predictor of survival in breast cancer N Engl J Med 2002 347 1999 2009 10.1056/NEJMoa021967 12490681

[B5] van't Veer LJ Dai H van de Vijver MJ He YD Hart AAM Mao M Peterse HL van der Kooy K Marton MJ Witteveen AT Schreiber GJ Kerkhoven RM Roberts C Linsley PS Bernards R Friend SH Gene expression profiling predicts clinical outcome of breast cancer Nature 2002 415 530 536 10.1038/415530a 11823860

[B6] Rhodes DR Yu J Shanker K Deshpande N Varambally R Ghosh D Barrette T Pandey A Chinnaiyan AM ONCOMINE: a cancer microarray database and integrated data-mining platform Neoplasia 2004 6 1 6 15068665 10.1016/s1476-5586(04)80047-2 PMC1635162

[B7] Wirapati P Sotiriou C Kunkel S Farmer P Pradervand S Haibe-Kains B Desmedt C Ignatiadis M Sengstag T Schütz F Goldstein DR Piccart M Delorenzi M Meta-analysis of gene expression profiles in breast cancer: toward a unified understanding of breast cancer subtyping and prognosis signatures Breast Cancer Res 2008 10 R65 10.1186/bcr2124 18662380 PMC2575538

[B8] Xu L Tan A Winslow R Geman D Merging microarray data from separate breast cancer studies provides a robust prognostic test BMC Bioinformatics 2008 9 125 10.1186/1471-2105-9-125 18304324 PMC2409450

[B9] Warnat P Eils R Brors B Cross-platform analysis of cancer microarray data improves gene expression based classification of phenotypes BMC Bioinformatics 2005 6 265 10.1186/1471-2105-6-265 16271137 PMC1312314

[B10] Bolstad BM Irizarry R Åstrand M Speed TP A comparison of normalization methods for high density oligonucleotide array data based on variance and bias Bioinformatics 2003 19 185 193 10.1093/bioinformatics/19.2.185 12538238

[B11] Benito M Parker J Du Q Wu J Xiang D Perou CM Marron JS Adjustment of systematic microarray data biases Bioinformatics 2004 20 105 114 10.1093/bioinformatics/btg385 14693816

[B12] Lander ES Array of hope Nat Genet 1999 21 3 4 9915492 10.1038/4427

[B13] Alter O Brown PO Botstein D Singular value decomposition for genome-wide expression data processing and modeling Proc Natl Acad Sci USA 2000 97 10101 10106 10963673 10.1073/pnas.97.18.10101 PMC27718

[B14] Jiang H Deng Y Chen H-S Tao L Sha Q Chen J Tsai C-J Zhang S Joint analysis of two microarray gene-expression data sets to select lung adenocarcinoma marker genes BMC Bioinformatics 2004 5 81 10.1186/1471-2105-5-81 15217521 PMC476733

[B15] Reyal F van Vliet MH Armstrong NJ Horlings HM de Visser KE Kok M Teschendorff AE Mook S van't Veer L Caldas C Salmon RJ van de Vijver MJ Wessels LFA A comprehensive analysis of prognostic signatures reveals the high predictive capacity of the proliferation, immune response and RNA splicing modules in breast cancer Breast Cancer Res 2008 10 R93 10.1186/bcr2192 19014521 PMC2656909

[B16] Acharya CR Hsu DS Anders CK Anguiano A Salter KH Walters KS Redman RC Tuchman SA Moylan CA Mukherjee S Barry WT Dressman HK Ginsburg GS Marcom KP Garman KS Lyman GH Nevins JR Potti A Gene expression signatures, clinicopathological features, and individualized therapy in breast cancer JAMA 2008 299 1574 1587 10.1001/jama.299.13.1574 18387932

[B17] Irizarry RA Hobbs B Collin F Beazer-Barclay YD Antonellis KJ Scherf U Speed TP Exploration, normalization, and summaries of high density oligonucleotide array probe level data Biostatistics 2003 4 249 264 10.1093/biostatistics/4.2.249 12925520

[B18] McCall MN Bolstad BM Irizarry RA Frozen robust multiarray analysis (fRMA) Biostatistics 2010 11 242 253 10.1093/biostatistics/kxp059 20097884 PMC2830579

[B19] Li C Wong WH Model-based analysis of oligonucleotide arrays: expression index computation and outlier detection Proc Natl Acad Sci USA 2001 98 31 36 10.1073/pnas.011404098 11134512 PMC14539

[B20] Li C Rabinovic A Adjusting batch effects in microarray expression data using empirical Bayes methods Biostatistics 2007 8 118 127 16632515 10.1093/biostatistics/kxj037

[B21] Chen C Grennan K Badner J Zhang D Gershon E Jin L Liu C Removing batch effects in analysis of expression microarray data: an evaluation of six batch adjustment methods PLoS One 2011 6 e17238 10.1371/journal.pone.0017238 21386892 PMC3046121

[B22] Chen QR Song YK Wei JS Bilke S Asgharzadeh S An integrated cross-platform prognosis study on neuroblastoma patients Genomics 2008 92 195 203 10.1016/j.ygeno.2008.05.014 18598751 PMC2562635

[B23] Zheng CH Huang DS Shang L Feature selection in independent component subspace for microarray data classification Neurocomputing 2006 69 2407 2410 10.1016/j.neucom.2006.02.006

[B24] Scherer A Batch Effects and Noise in Microarray Experiments: Sources and Solutions 2009 1 Wiley

[B25] Subramanian A Tamayo P Mootha VK Mukherjee S Ebert BL Gillette MA Paulovich A Pomeroy SL Golub TR Lander ES Mesirov JP Gene set enrichment analysis: a knowledge-based approach for interpreting genome-wide expression profiles Proc Natl Acad Sci USA 2005 102 15545 15550 10.1073/pnas.0506580102 16199517 PMC1239896

[B26] Brugger D Bogdan M Rosenstiel W Automatic cluster detection in Kohonen's SOM IEEE Trans Neural Netw 2008 19 442 459 18334364 10.1109/TNN.2007.909556

[B27] Kim S-Y Volsky DJ PAGE: parametric analysis of gene set enrichment BMC Bioinformatics 2005 6 144 144 10.1186/1471-2105-6-144 15941488 PMC1183189

[B28] Foekens JA Atkins D Zhang Y Sweep FCGJ Harbeck N Paradiso A Cufer T Sieuwerts AM Talantov D Span PN Tjan-Heijnen VCG Zito AF Specht K Hoefler H Golouh R Schittulli F Schmitt M Beex LVAM Klijn JGM Wang Y Multicenter validation of a gene expression-based prognostic signature in lymph node-negative primary breast cancer J Clin Oncol 2006 24 1665 1671 10.1200/JCO.2005.03.9115 16505412

[B29] Yasrebi H Sperisen P Praz V Bucher P Can survival prediction be improved by merging gene expression data sets? PLoS One 2009 4 e7431 10.1371/journal.pone.0007431 19851466 PMC2761544

[B30] Wang Y Klijn JGM Zhang Y Sieuwerts AM Look MP Yang F Talantov D Timmermans M Meijer-van Gelder ME Yu J Jatkoe T Berns EMJJ Atkins D Foekens JA Gene-expression profiles to predict distant metastasis of lymph-node-negative primary breast cancer Lancet 2005 365 671 679 15721472 10.1016/S0140-6736(05)17947-1

[B31] Guyon I Weston J Barnhill S Vapnik V Gene selection for cancer classification using support vector machines Machine Learning 2002 46 389 422 10.1023/A:1012487302797

[B32] Huang DS Zheng CH Independent component analysis-based penalized discriminant method for tumor classification using gene expression data Bioinformatics 2006 22 15 1855 1862 10.1093/bioinformatics/btl190 16709589

[B33] Zheng CH Huang DS Kong XZ Zhao XM Gene expression data classification using consensus independent component analysis Genomics Proteomics Bioinformatics 2008 6 74 82 10.1016/S1672-0229(08)60022-4 18973863 PMC5054104

[B34] Tusher VG Tibshirani R Chu G Significance analysis of microarrays applied to the ionizing radiation response Proc Natl Acad Sci USA 2001 98 5116 5121 10.1073/pnas.091062498 11309499 PMC33173

[B35] MeV: MultiExperiment Viewer | Part of the TM4 Microarray Software Suite http://www.tm4.org/mev/

[B36] Wang H-Q Wong H-S Huang DS Shu J Extracting gene regulation information for cancer classification Pattern Recognition 2007 40 3379 3392 10.1016/j.patcog.2007.04.007

